# Case Report: Iatrogenic trauma of the bladder due to long-term unidentified intrauterine device malposition inside the bladder with rectovesical fistula

**DOI:** 10.12688/f1000research.136351.1

**Published:** 2023-10-20

**Authors:** Ahmad Agil, Tjahjodjati Tjahjodjati, Nur Atik, Dedi Rachmadi, Tengku Tania Zahrina

**Affiliations:** 1 Department of Urology, Universitas Padjadjaran, Bandung, West Java, 40132, Indonesia; 2 Department of Biomedical Science, Universitas Padjadjaran, Bandung, West Java, Indonesia; 3 Department of Pediatrics, Universitas Padjadjaran, Bandung, West Java, Indonesia

**Keywords:** intrauterine device, cystoscopy, vesicolithiasis, iatrogenic bladder trauma

## Abstract

According to reports, there are 1.9–3.6 incidences of IUD migration and uterine perforation for every 1000 IUD insertions. It is important to note that bladder perforation caused by a misplaced IUD is uncommon and is thought to happen most frequently during insertion. Here, we describe a patient who presented with symptoms related to the migration of IUD to the bladder. It is feasible to draw the conclusion that the cystoscopy technique should be taken into consideration as a suitable therapy option for such injuries in this organ. When a problem cannot be effectively treated by cystoscopy alone, laparotomy should be considered.

## Introduction

The intrauterine device (IUD), a small T-shaped piece of plastic that is used as a form of contraception, has the potential to perforate the uterus and spread to the pelvic or abdominal organs. According to reports, there are 1.9–3.6 incidences of IUD migration and uterine perforation for every 1000 IUD insertions. It is important to note that bladder perforation caused by a misplaced IUD is uncommon and is thought to happen most frequently during insertion. According to the literature, there are three ways to remove an IUD that has migrated to the lower urinary tract: a laparoscopy, open surgery, or a cystoscopy. ^1^

Although potential causes of ectopic IUD have been proposed, no official study has been done on the topic due to the rarity of the occurrences. ^2^ After parturition, a weak uterine wall combined with an ill-advised early implantation may cause the IUD to become entrenched in the uterine wall and eventually shift; ectopic displacement of the IUD may be caused by aberrant morphology and the uterus’ regular contractions; The material and shape of IUDs are continuously optimized and improved to lessen the side effects of IUD placement, but unsuitable material and shape may cause chronic incision to the uterine wall during uterine contraction, ultimately causing the IUD to shift and embed in the posterior wall of the bladder.

Procedures carried out in or near the retroperitoneal abdominal space or pelvis has the potential to result in iatrogenic harm to the urinary tract, including the kidneys, ureters, bladder, and urethra. Discussions of these injuries are frequently directed toward specialists like urologists, obstetricians, gynecologists, and general surgeons whose procedures are most frequently implicated in iatrogenic urinary tract injuries. ^3^

Iatrogenic bladder injury should be recognized as soon as it happens. According to a study by Adelman *et al.*, of the 100 cases that were detected in studies during the past 10 years, more than 80% were discovered throughout the course of the treatment. In addition to being able to see the injured tissue directly, external bladder traumas may also be suspected if urine was discovered in the operating room, air was detected in the collection bag for the Foley catheter, or the Foley catheter itself was visible. Iatrogenic internal bladder injuries may cause new symptoms to appear such as abdominal bloating, trouble sustaining bladder distension with infused fluid, and the ability to see urine outside the bladder. ^4^

Although surgical repair of intraperitoneal bladder injuries is often accomplished by a laparotomy, little is known about minimally invasive therapies in this clinical situation. Improved view of the pelvic organs, earlier return to daily activities, reduced bleeding, postoperative pain, intraabdominal adhesions, danger of incisional hernias, and duration of hospital stay and incapacity are the advantages of the laparoscopic technique. ^5^

Here, we describe a patient who presented with symptoms related to the migration of IUD to the bladder.

## Case report

This study was performed at Hasan Sadikin General Hospital, Bandung, Indonesia in July 2022. Informed consent for the publication of this article was obtained from the patient.

**Figure 1.**
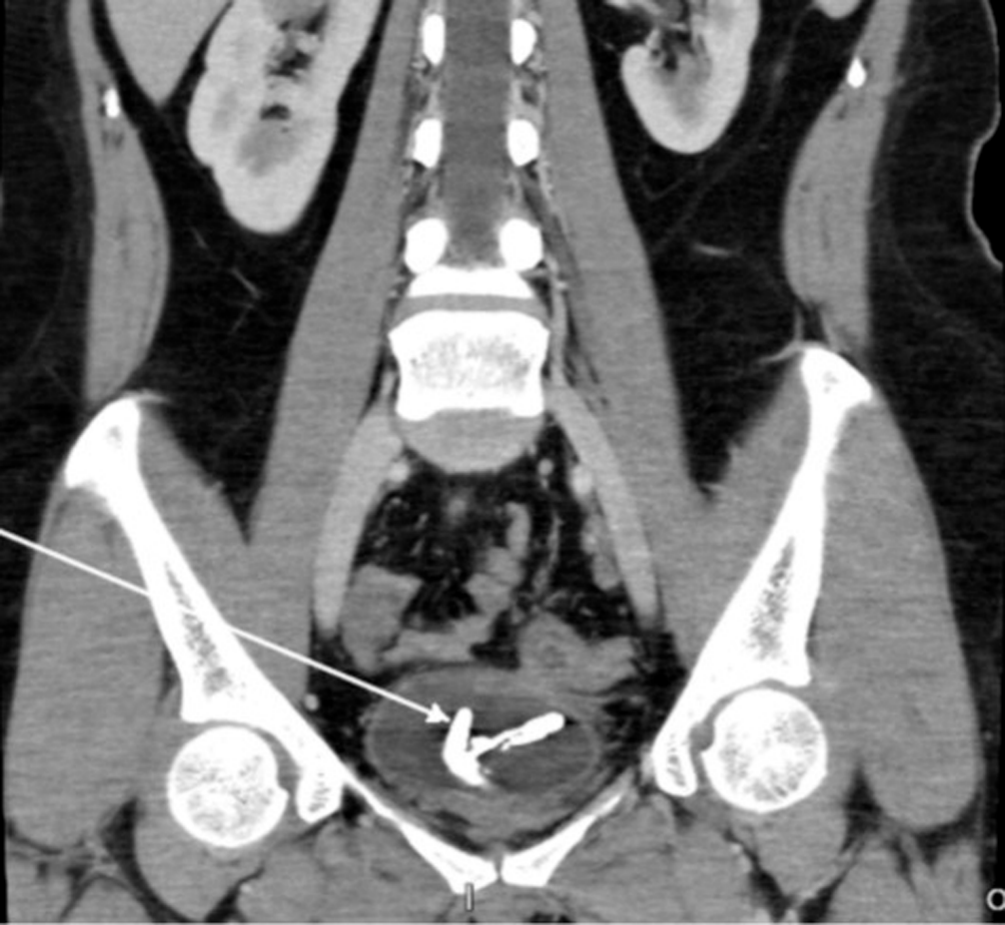
CT scan revealed an encrustation of IUD in the bladder.

**Figure 2.**
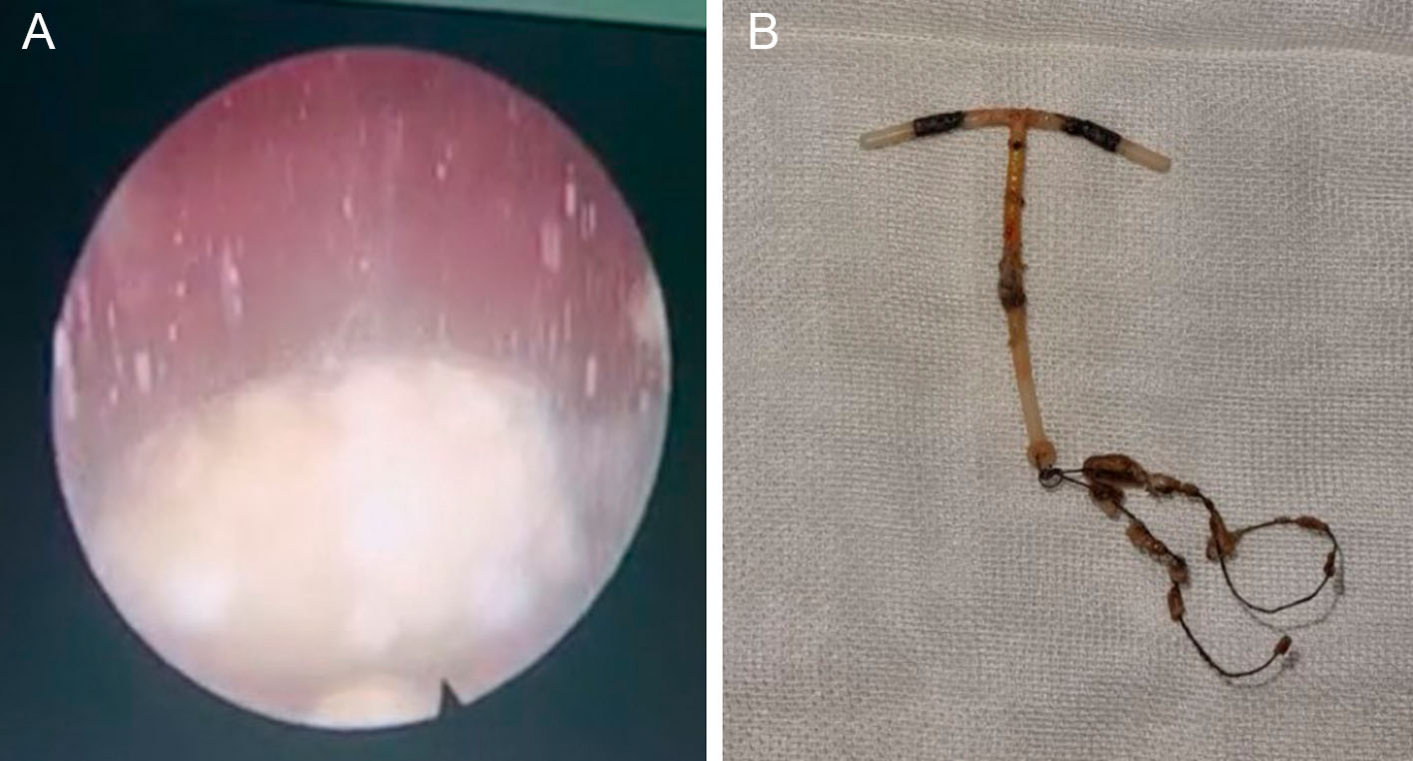
Cystoscopy, lithotripsy and IUD evacuation procedure.

A 36-year-old woman presented with lower urinary tract obstruction symptoms. An abdominal CT scan revealed an encrustation of corpus alienum in the bladder, due to malposition of IUD copper T ( Figure 1). The patient underwent cystoscopy + lithotripsy + IUD copper evacuation ( Figure 2). Intraoperative findings revealed there was left posterolateral rectovesicula fistule. Clinically, no digestive remainders were found in the urine. CT scan examination also did not find any signs leading to rectovesicula fistule.

The patient had the IUD implanted for six years before the case. There was a history of pregnancy, but the patient underwent curettage due to abortion. During curettage, there was no IUD to be found on the uterus. The patient then was planned to undergo exploratory laparotomy and fistule repair in a joint procedure with digestive surgeon.

Most authors agree that having an IUD placed by a gynecologist is crucial for preventing perforation. However, gynecologists also have been known to insert IUDs that migrate. ^6^ In the present case, the IUD was inserted by a midwife. Additionally, the vaginal speculum used for IUD implantation can cause tissue injury and infection, which can result in adhesions that make the uterus more likely to be punctured. ^7^

Macroscopic hematuria, abdominal or suprapubic discomfort, the inability to urinate, and oliguria are all indications of bladder damage. These signs and symptoms typically occur within the first 48 hours following surgery for a thermal injury or up to 10–14 days later. Because of the aberrant spike in serum creatinine levels brought on by the substance’s reabsorption into the urine through the peritoneal membrane, biochemical profiles are used to diagnose this kind of damage. ^8^ However, in our case, the signs and symptoms of vesicolithiasis were more dominant due to the encrustation of IUD in the bladder.

Cystoscopy and imaging, such as plain X-rays, computed tomography, and ultrasound, offer significant diagnostic assistance and are crucial in determining the appropriate surgical techniques and approaches. ^9^ CT scan played an important role in identifying the ectopic IUD in our case, but failed to detect the rectovesica fistule.

*Actinomyces* infections, as is well known, can also cause perforation of the uterus. In the presence of an IUD, *Actinomyces* infection can frequently arise. ^10^ Another noteworthy problem is the increased likelihood of IUD migration in women who give birth while their IUD is still in place. The uterus is more prone to perforation because of the hypoestrogenemia-induced shrinkage of the uterus and thinned uterine walls during the postpartum and breastfeeding periods. ^6^

Cystoscopy and lithotripsy were used in our case to evacuate the IUD and demolish the calculus formed in the bladder. At first, there was no intention to close the injury as there was no manifestation of the trauma of the bladder wall due to primary closure. As the rectovesicula fistule was found intraoperatively, the laparotomy became mandatory.

Absorbable suture should be used for cystotomy repair, or surgical suture used to seal a bladder damage, to prevent producing a nidus that encourages the development of kidney stones. Additionally, it can be carried out using a single-layer or two-layer approach, interrupted or continuous. ^8^ For two weeks, urinary diversion with a Foley catheter for continuous drainage should be kept up. ^11^

Only a small number of case reports have described laparoscopic IUD evacuation surgery. ^12^ Additionally, open surgery or laparoscopic partial cystectomies have been successful procedures for some individuals. ^13^ One instance reported by Atakan *et al*. required a suprapubic cystotomy. ^14^ These results imply that ectopic IUDs lodged in the bladder wall can be treated with both open and laparoscopic operations. However, there are further approaches that might be investigated.

This report contains some flaws. This case examined a patient whose IUD became stuck in the bladder wall and who also had a calculus that had been treated at one hospital. To compare open versus laparoscopic operations, the number of patients was insufficient. The decision between laparoscopy and open surgery should be made specifically for each instance because of the uniqueness of the problem, the large variety of IUDs on the market, and the conditions unique to each patient. The IUD develops a calculus when it comes into touch with urine, according to our observation.

## Conclusions

IUD migration to the bladder should be suspected if bladder stones are observed, especially in women who have given birth while wearing an IUD, urinary tract infections that are resistant to treatment, and symptoms including dyspareunia and vaginal discharge. The conclusion can be drawn that the cystoscopy technique should be taken into consideration as a suitable therapy option for such injuries in this organ. When there is a problem that cannot be effectively treated by cystoscopy alone, laparotomy should be considered.

### Patient consent

Written informed consent for publication of their clinical details and clinical images was obtained from the patient.

## Data Availability

All data underlying the results are available as part of the article and no additional source data are required.
