# Case Report: Iatrogenic trauma of the bladder due to long-term unidentified intrauterine device malposition inside the bladder with rectovesical fistula

**DOI:** 10.12688/f1000research.136351.2

**Published:** 2024-01-05

**Authors:** Ahmad Agil, Tjahjodjati Tjahjodjati, Nur Atik, Dedi Rachmadi, Tengku Tania Zahrina

**Affiliations:** 1 Department of Urology, Universitas Padjadjaran, Bandung, West Java, 40132, Indonesia; 2 Department of Biomedical Science, Universitas Padjadjaran, Bandung, West Java, Indonesia; 3 Department of Pediatrics, Universitas Padjadjaran, Bandung, West Java, Indonesia

**Keywords:** intrauterine device, cystoscopy, vesicolithiasis, iatrogenic bladder trauma

## Abstract

According to reports, there are 1.9–3.6 incidences of IUD migration and uterine perforation for every 1000 IUD insertions. It is important to note that bladder perforation caused by a misplaced IUD is uncommon and is thought to happen most frequently during insertion. Here, we describe a patient who presented with symptoms related to the malposition of IUD inside the bladder. It is feasible to draw the conclusion that the cystoscopy technique should be taken into consideration as a suitable therapy option for such injuries in this organ. When a problem cannot be effectively treated by cystoscopy alone, laparotomy should be considered.

## Introduction

The intrauterine device (IUD), a small T-shaped piece of plastic that is used as a form of contraception, has the potential to perforate the uterus and spread to the pelvic or abdominal organs. According to reports, there are 1.9–3.6 incidences of IUD migration and uterine perforation for every 1000 IUD insertions. It is important to note that bladder perforation caused by a misplaced IUD is uncommon and is thought to happen most frequently during insertion. According to the literature, there are three ways to remove an IUD that has migrated to the lower urinary tract: a laparoscopy, open surgery, or a cystoscopy. ^1^

Although potential causes of ectopic IUD have been proposed, no official study has been done on the topic due to the rarity of the occurrences. ^2^ Following childbirth, a weakened uterine wall, coupled with an inadvisable early implantation, can lead to the IUD becoming embedded in the uterine wall and eventually shifting. Ectopic displacement of the IUD can result from abnormal morphology and the regular contractions of the uterus. While the material and shape of IUDs are continually refined to minimize side effects, improper material and shape can lead to persistent abrasion in the uterine wall during contractions, ultimately causing the IUD to shift and embed in the posterior wall of the bladder.

Procedures carried out in or near the retroperitoneal abdominal space or pelvis has the potential to result in iatrogenic harm to the urinary tract, including the kidneys, ureters, bladder, and urethra. Discussions of these injuries are frequently directed toward specialists like urologists, obstetricians, gynecologists, and general surgeons whose procedures are most frequently implicated in iatrogenic urinary tract injuries. ^3^

Iatrogenic bladder injury should be recognized as soon as it happens. According to a study by Adelman *et al.*, of the 100 cases that were detected in studies during the past 10 years, more than 80% were discovered throughout the course of the treatment. In addition to being able to see the injured tissue directly, external bladder traumas may also be suspected if urine was discovered in the operating room, air was detected in the collection bag for the Foley catheter, or the Foley catheter itself was visible. Iatrogenic internal bladder injuries may cause new symptoms to appear such as abdominal bloating and trouble sustaining bladder distension with infused fluid. ^4^

Although surgical repair of intraperitoneal bladder injuries is often accomplished by a laparotomy, little is known about minimally invasive therapies in this clinical situation. Improved view of the pelvic organs, earlier return to daily activities, reduced bleeding, postoperative pain, intraabdominal adhesions, danger of incisional hernias, and duration of hospital stay and incapacity are the advantages of the laparoscopic technique. ^5^

Here, we describe a patient who presented with symptoms related to the malposition of IUD inside the bladder.

## Case report

This study was performed at Hasan Sadikin General Hospital, Bandung, Indonesia in July 2022. Informed consent for the publication of this article was obtained from the patient.

**Figure 1.**
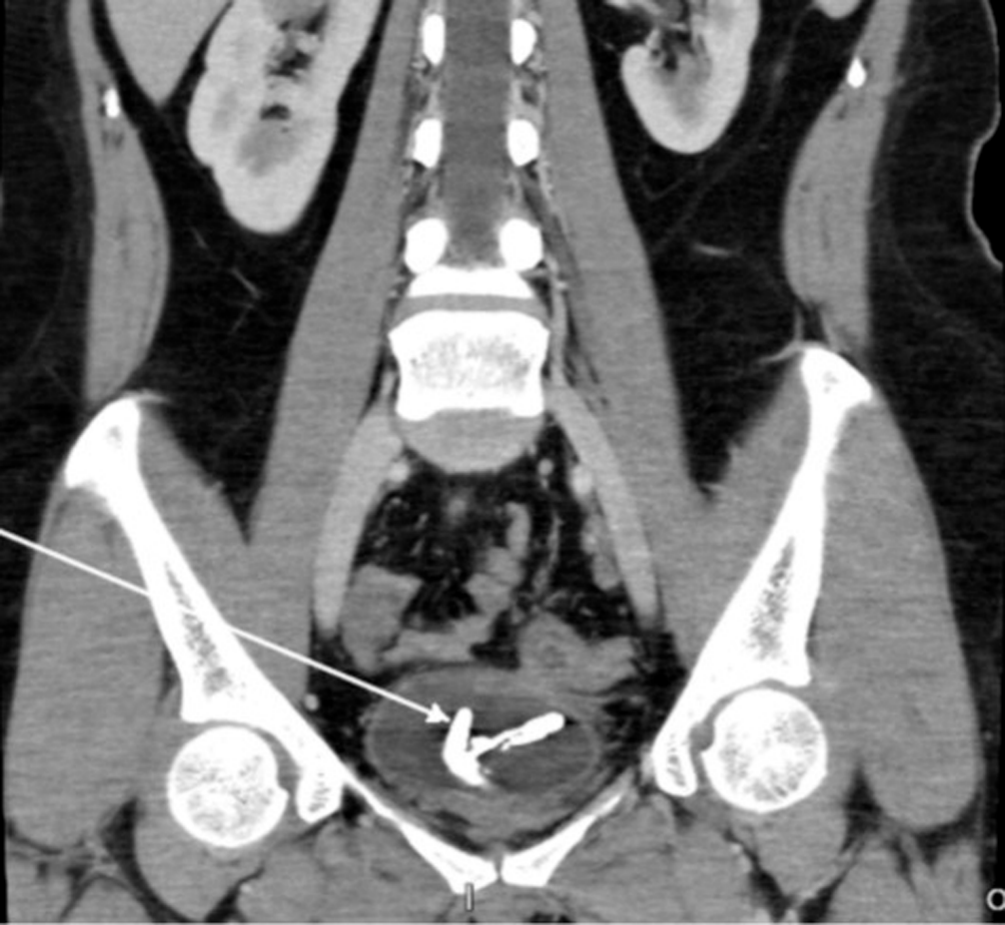
CT scan revealed an encrustation of IUD in the bladder.

**Figure 2.**
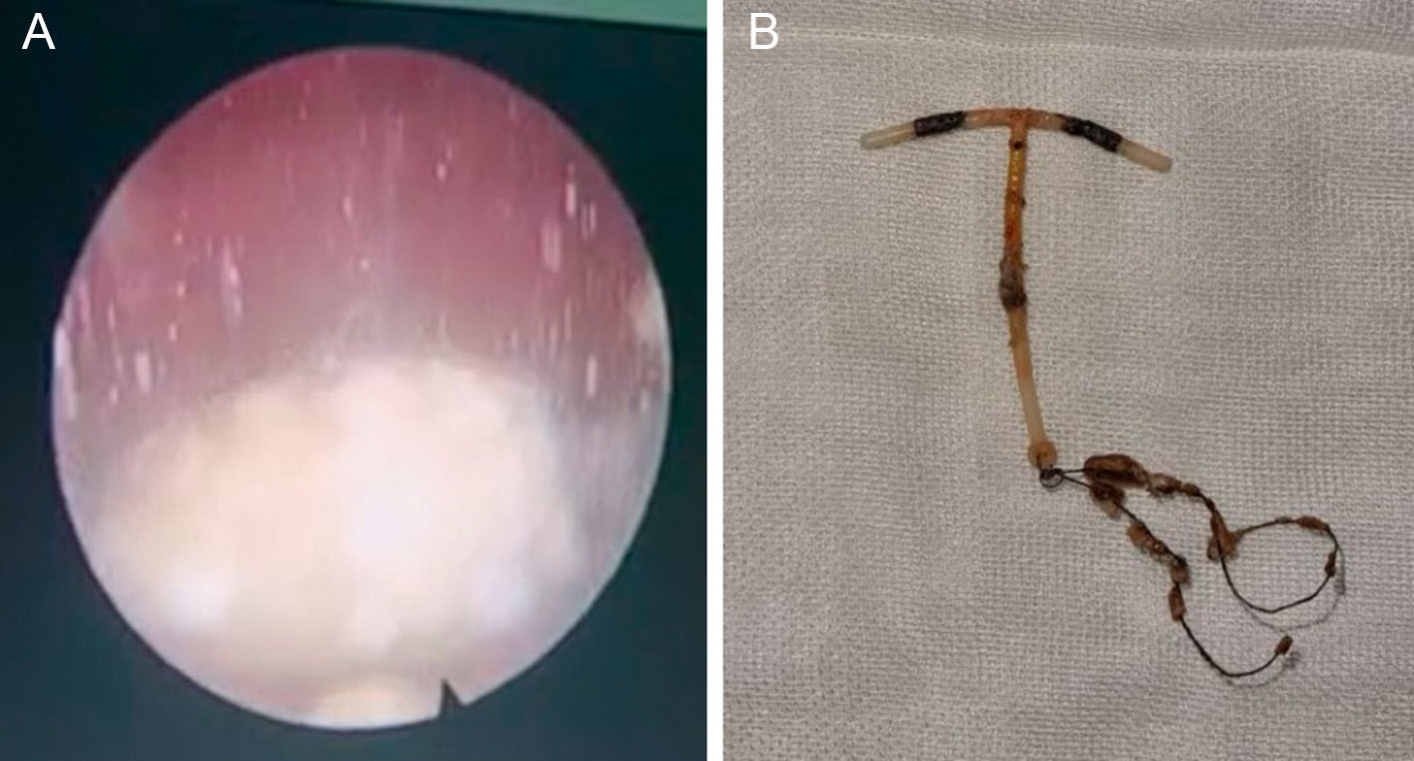
Cystoscopy, lithotripsy and IUD evacuation procedure.

A 36-year-old woman presented with lower urinary tract symptoms, such as dysuria and intermittent cloudy urine for several months. There were no sign of fecaluria and pneumaturia, and from urinalysis there was no sign of enteric content material inside the urine. An abdominal CT scan revealed an encrustation of corpus alienum in the bladder, due to malposition of IUD copper T, but no sign of rectovesical fistulae ( Figure 1). The patient underwent cystoscopy + lithotripsy + IUD copper evacuation ( Figure 2). Intraoperative findings revealed there was left posterolateral rectovesical fistulae.

The patient had the IUD implanted for six years before the case. There was a history of pregnancy, but the patient underwent curettage due to abortion. During curettage, there was no IUD could be located in the uterus. The patient then was planned to undergo exploratory laparotomy and fistulae repair in a joint procedure with digestive surgeon.

Most authors agree that having an IUD placed by a gynecologist is crucial for preventing perforation. Nevertheless, gynaecologists are also known to insert IUD that could also migrate. ^6^ In the present case, the IUD was inserted by a midwife. Additionally, the vaginal speculum used for IUD implantation can cause tissue injury and infection, which can result in adhesions that make the uterus more likely to be punctured. ^7^ Another potential issue could be the misplacement of the IUD during insertion. It should be inserted into the uterus, but there is a risk of it being mistakenly placed into the bladder.

Macroscopic hematuria, abdominal or suprapubic discomfort, the inability to urinate, and oliguria are all indications of bladder damage. These signs and symptoms typically occur within the first 48 hours following surgery for a thermal injury or up to 10–14 days later. Because of the aberrant spike in serum creatinine levels brought on by the substance’s reabsorption into the urine through the peritoneal membrane, biochemical profiles are used to diagnose this kind of damage. ^8^ However, in our case, the signs and symptoms of bladder stone were more dominant due to the encrustation of IUD in the bladder.

Cystoscopy and imaging, such as plain X-rays, computed tomography, and ultrasound, offer significant diagnostic assistance and are crucial in determining the appropriate surgical techniques and approaches. ^9^ CT scan played an important role in identifying the ectopic IUD in our case, but failed to detect the rectovesical fistulae.

*Actinomyces* infections, as is well known, can also cause perforation of the uterus. In the presence of an IUD, *Actinomyces* infection can frequently arise. ^10^ Another noteworthy problem is the increased likelihood of IUD migration in women who give birth while their IUD is still in place. The uterus is more prone to perforation because of the hypoestrogenemia-induced shrinkage of the uterus and thinned uterine walls during the postpartum and breastfeeding periods. ^6^

Cystoscopy and lithotripsy were used in our case to evacuate the IUD and demolish the calculus formed in the bladder. At first, there was no intention to close the injury as there was no manifestation of the trauma of the bladder wall due to primary closure. As the rectovesical fistulae was found intraoperatively, the laparotomy became mandatory. We have already consulted the patient to digestive surgeon intraoperatively and advised to undergo colostomy diversion with concomitant bladder repair, but unfortunately, she refused the action plan.

Technically if the bladder repair was done, absorbable suture should be used to seal a bladder damage, to prevent producing a nidus that encourages the development of bladder stones. Additionally, it can be carried out using a single-layer or two-layer approach, interrupted or continuous. ^8^ For two weeks, urinary diversion with a Foley catheter for continuous drainage should be kept up. ^11^

Only a small number of case reports have described laparoscopic IUD evacuation surgery for intraabdominal migration. ^12^ Additionally, open surgery or laparoscopy have been successful procedures for some individuals for the case of intraabdominal migration. ^13^ These results imply that ectopic IUD with intraabdominal migration can be treated with both open and laparoscopic surgery. One instance reported by Atakan *et al.* only required cystoscopy for intravesical migration. ^14^

After the treatment, in the first follow-up, the patient was in a good condition, had no dysuria or abdominal pain, and had clear urine. The patient was consulted again to the digestive surgeon, but unfortunately, she was lost to follow-up.

However, this report contains some weakness. First, the patient had been treated before at another hospital with various antibiotics. Second, we cannot track the patient’s condition to its conclusion as the patient has lost follow-up.

## Conclusions

IUD malposition inside the bladder should be suspected if bladder stones are observed, particularly in women who have given birth while using an IUD. Other indicators include urinary tract infections resistant to treatment, as well as symptoms such as dyspareunia and vaginal discharge. It can be inferred that cystoscopy is a viable therapeutic option for addressing injuries in this organ. If a problem proves challenging for cystoscopy alone, open surgery should be considered.

### Patient consent

Written informed consent for publication of their clinical details and clinical images was obtained from the patient.

## Data Availability

All data underlying the results are available as part of the article and no additional source data are required.
