# Supplementary material for: Case Report: Iatrogenic trauma of the bladder due to long-term unidentified intrauterine device malposition inside the bladder with rectovesical fistula
Source: F1000Res. 2024 Jan 5;12:1390. Originally published 2023 Oct 20. [Version 2] doi: 10.12688/f1000research.136351.2 (PMC10905013; doi:10.12688/f1000research.136351.2)
Supplement: Peer review history

# Reviewer report: Reviewer response for version 2

**DOI:** 10.5256/f1000research.160269.r235882

**Published:** 2024-02-13

**Authors:** Prahara Yuri

**Affiliations:** 1 Division of Urology, Department of Surgery, Faculty of Medicine, Public Health and Nursing, Dr. Sardjito Hospital, Universitas Gadjah Mada, Yogyakarta, Indonesia

Thank you for your explanation and revisions, all statements fulfill the requirement.

Are enough details provided of any physical examination and diagnostic tests, treatment given and outcomes?

Partly

Is the case presented with sufficient detail to be useful for other practitioners?

Partly

Is sufficient discussion included of the importance of the findings and their relevance to future understanding of disease processes, diagnosis or treatment?

Yes

Is the background of the case’s history and progression described in sufficient detail?

Yes

Reviewer Expertise:

Pediatric urology, reconstruction, endourology, uro-andrology and laparoscopic urology

I confirm that I have read this submission and believe that I have an appropriate level of expertise to confirm that it is of an acceptable scientific standard.

# Reviewer report: Reviewer response for version 1

**DOI:** 10.5256/f1000research.149490.r218419

**Published:** 2023-11-20

**Authors:** Prahara Yuri

**Affiliations:** 1 Division of Urology, Department of Surgery, Faculty of Medicine, Public Health and Nursing, Dr. Sardjito Hospital, Universitas Gadjah Mada, Yogyakarta, Indonesia

Thank you for submitting your valuable case in F1000 research. Some questions should be addressed:

1. When did you performed the fistula repair procedure? And explain the patient's follow up?
2. Did the IUD evacuation and fistula repair performed in different time or in the same time? Please explain why?
3. What was the different of your case comparing to others?
4. What were the possibility causes of IUD migration in your case? 

Are enough details provided of any physical examination and diagnostic tests, treatment given and outcomes?

Partly

Is the case presented with sufficient detail to be useful for other practitioners?

Partly

Is sufficient discussion included of the importance of the findings and their relevance to future understanding of disease processes, diagnosis or treatment?

Yes

Is the background of the case’s history and progression described in sufficient detail?

Yes

Reviewer Expertise:

Pediatric urology, reconstruction, endourology, uro-andrology and laparoscopic urology

I confirm that I have read this submission and believe that I have an appropriate level of expertise to confirm that it is of an acceptable scientific standard, however I have significant reservations, as outlined above.

# Response

**Published:** 2023-11-19

**Authors:** Ahmad Agil

**Affiliations:** Urology, Universitas Padjadjaran, Bandung, Jawa Barat, Indonesia

In this case, the only preoperative symptom that appears is urination that smells. Then, the preoperative CT scan results did not detect a fistula. We also did not predict that the patient's condition would be so severe that a fistula would occur. However, during the intraoperative procedure, we discovered a fistula. This is one of the unique things in our case.

 Best regards,

 Ahmad Agil

# Response

**Published:** 2023-11-24

**Authors:** Ahmad Agil

**Affiliations:** Urology, Universitas Padjadjaran, Bandung, Jawa Barat, Indonesia

1. After the treatment, a post-operation follow-up was completed. In the first follow-up, the patient was in good condition, had no dysuria or abdominal pain, and had clear urine. The patient was planned to be consulted by the digestive surgeon, but unfortunately, the patient was lost to follow-up.

 2. IUD evacuation and fistula repair were not performed at the same time, because when consulted for digestive surgery the patient was advised to undergo colostomy diversion. However, the patient refused the action plan.

 3. This case is a rare case where IUD malposition can cause complications including fistulas. This condition was also not detected during the previous examination because no significant symptoms were found. The patient only complained of dysuria and intermittent cloudy urination.

 4. In this case the cause was not IUD migration but rather an error in placing the IUD during installation by the midwife. The patient said that after 1 year of installing the IUD it was found that the patient was pregnant and when checking, the IUD was not found during delivery.

# Reviewer report: Reviewer response for version 1

**DOI:** 10.5256/f1000research.149490.r220884

**Published:** 2023-11-20

**Authors:** Pande Made Wisnu Tirtayasa

**Affiliations:** 1 Department of Urology, Faculty of Medicine, Universitas Udayana Teaching Hospital, Universitas Udayana, Jimbaran, Bali, Indonesia

The title suggests that the patient also suffered from a rectovesical fistula. However, from the case explanation, no single examination led to the proof of a rectovesical fistula. Please consider adding additional statements/sentences that explain how the authors diagnose the rectovesical fistula and the management regarding it.

 In general, this study has covered all requirements to be indexed as a case report study.

Are enough details provided of any physical examination and diagnostic tests, treatment given and outcomes?

Partly

Is the case presented with sufficient detail to be useful for other practitioners?

Yes

Is sufficient discussion included of the importance of the findings and their relevance to future understanding of disease processes, diagnosis or treatment?

Yes

Is the background of the case’s history and progression described in sufficient detail?

Yes

Reviewer Expertise:

Urology, kidney transplant, pediatric urology, biology molecular, immunology

I confirm that I have read this submission and believe that I have an appropriate level of expertise to confirm that it is of an acceptable scientific standard.
